# Optimal Control Analysis of Pneumonia and Meningitis Coinfection

**DOI:** 10.1155/2019/2658971

**Published:** 2019-09-22

**Authors:** Getachew Teshome Tilahun

**Affiliations:** Haramaya University, Department of Mathematics, Dire Dawa, Ethiopia

## Abstract

In this paper, we proposed a deterministic model of pneumonia-meningitis coinfection. We used a system of seven ordinary differential equations. Firstly, the qualitative behaviours of the model such as positivity of the solution, existence of the solution, the equilibrium points, basic reproduction number, analysis of equilibrium points, and sensitivity analysis are studied. The disease-free equilibrium is locally asymptotically stable if the basic reproduction number is kept less than unity, and conditions for global stability are established. Then, the basic model is extended to optimal control by incorporating four control interventions, such as prevention of pneumonia as well as meningitis and also treatment of pneumonia and meningitis diseases. The optimality system is obtained by using Pontryagin's maximum principle. For simulation of the optimality system, we proposed five strategies to check the effect of the controls. First, we consider prevention only for both diseases, and the result shows that applying prevention control has a great impact in bringing down the expansion of pneumonia, meningitis, and their coinfection in the specified period of time. The other strategies are prevention effort for pneumonia and treatment effort for meningitis, prevention effort for meningitis and treatment effort for pneumonia, treatment effort for both diseases, and using all interventions. We obtained that each of the listed strategies is effective in minimizing the expansion of pneumonia-only, meningitis-only, and coinfectious population in the specified period of time.

## 1. Introduction

Pneumonia, which can be categorized as one of the airborne diseases, claims for the death of millions of human beings through inhaling pathogenic organism, mainly *Streptococcus pneumoniae* [1]. These bacteria are also responsible for the cause of other diseases, such as meningitis, ear infections, and sinus infections. Pneumonia can affect human beings of all ages, from children to the elderly, and it becomes dangerous when the immunity level is lowered, as well as when it is coinfected with other diseases like meningitis [2]. Meningitis, an infection which covers the brain and spinal cord, is caused by both bacteria and virus. Bacterial infection of meningitis is the most common one, particularly, *Streptococcus pneumoniae*, *Haemophilus influenzae*, and *Neisseria meningitidis* are responsible for 80% cause of meningitis [3]. To control these diseases, a lot of scholars proposed different methods. In this aspect, mathematical models played a great role in proposing controlling strategies. Several scholars proposed different models to describe the dynamics of infectious diseases in the community. Some of them [4–8] proposed a mathematical model of pneumonia only, and the others [9–11] proposed a mathematical model of meningitis only. Few scholars like Tilahun et al. [12] proposed a mathematical model of pneumonia and typhoid fever coinfection using optimal control strategies. Moreover, Onyinge et al. and Akinyi et al. [13, 14] developed a mathematical model for coinfection of pneumonia with malaria and HIV. More recently, Tilahun [15] proposed a mathematical model of pneumonia and meningitis and investigated their coinfection using an SIR approach. However, to the best of our knowledge, no one has proposed a mathematical model by incorporating optimal control strategies for coinfection of pneumonia and meningitis. Therefore, this work is devoted in fulfilling this gap.

This paper is organized as follows.  Section 2 presents the description of the model. Qualitative behaviour of the model is discussed in Section 3. In Section 4, the basic model is extended to optimal control analysis. In Section 5, numerical simulation of the optimality system is presented. A brief discussion and conclusion are presented in Section 6.

## 2. Description of the Model

**Figure 1:**
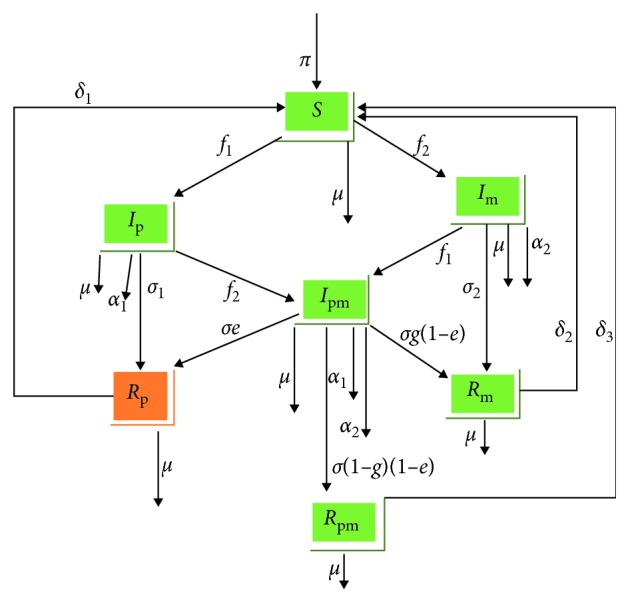
Flow diagram of the model.

In this section, a deterministic mathematical model of pneumonia-meningitis coinfection is presented. The model is proposed using seven compartments with total population size denoted by *N*(*t*). The compartment that has individuals who are healthy but able to be infected is denoted by *S*(*t*). Individuals that are affected by pneumonia and can transmit the disease to others are denoted by *I*_p_(*t*). Similarly, meningitis-infected individuals' compartment is denoted by *I*_m_(*t*), and coinfectious individuals' compartment is represented by *I*_pm_(*t*). Additionally, recovered /removed compartments from pneumonia, meningitis, and coinfection of both diseases are denoted by *R*_p_(*t*), *R*_m_(*t*), and *R*_pm_(*t*), respectively. Then, the total population is *N*(*t*)=*S*(*t*)+*I*_p_(*t*)+*I*_m_(*t*)+*I*_pm_(*t*)+*R*_p_(*t*)+*R*_m_(*t*)+*R*_pm_(*t*). Susceptible compartment increase by recruitment rate of *π* and also from Pneumonia recovered compartment with rate of δ_1_, meningitis recovered compartment with rate of δ_2_ and from co-infectious recovered compartment with rate of δ_3_. Force of infection of pneumonia and meningitis is *f*_1_=(*a*(*I*_p_(*t*)+*I*_pm_(*t*)))/*N* and *f*_2_=(*b*(*I*_m_(*t*)+*I*_pm_(*t*)))/*N*, respectively, where *a* is the contact rate of pneumonia and *b* is the contact rate of meningitis. Pneumonia-only recovered compartment is increased due to the recovery rate of pneumonia denoted by *σ*_1_, and meningitis-only and coinfectious recovered compartments increase their number with a rate of recovery of *σ*_2_ and *σ*, respectively. In the coinfectious recovered/removed compartment, individuals either recovered only from pneumonia, meningitis, or from both diseases with a probability of *σ*(1 − *e*), *σg*(1 − *e*) or *σ*(1 − *g*)(1 − *e*), respectively, where sigma, e, and g are any number between zero and one. The natural death rate is denoted by *μ* and pneumonia-causing death rate and meningitis-causing death rate are represented by *α*_1_ and *α*_2_, respectively. All parameters described in this model are assumed as nonnegative. The above description of the model is plotted in Figure 1.

From the flow diagram (Figure 1) of the model, the following system of differential equations is obtained:

(1) $$\begin{array}{c} \left\{\begin{array}{c} \frac{dS\left(t\right)}{dt}=\pi +\delta_{1}R_{\text{p}}\left(t\right)+\delta_{2}R_{\text{m}}\left(t\right)+\delta_{3}R_{\text{pm}}\left(t\right)-\left(f_{1}+f_{2}+\mu \right)S\left(t\right),\\ \frac{dI_{\text{p}}\left(t\right)}{dt}=f_{1}S\left(t\right)-\left(f_{2}+\sigma_{1}+\alpha_{1}+\mu \right)I_{\text{p}}\left(t\right),\\ \frac{dI_{\text{m}}\left(t\right)}{dt}=f_{2}S\left(t\right)-\left(f_{1}+\sigma_{2}+\alpha_{2}+\mu \right)I_{\text{m}}\left(t\right),\\ \frac{dI_{\text{pm}}\left(t\right)}{dt}=f_{2}I_{\text{p}}\left(t\right)+f_{1}I_{\text{m}}\left(t\right)-\left(\sigma +\alpha_{1}+\alpha_{2}+\mu \right)I_{\text{pm}}\left(t\right),\\ \frac{dR_{\text{p}}\left(t\right)}{dt}=\sigma_{1}I_{\text{p}}\left(t\right)+\sigma eI_{\text{pm}}\left(t\right)-\left(\delta_{1}+\mu \right)R_{\text{p}}\left(t\right),\\ \frac{dR_{\text{m}}\left(t\right)}{dt}=\sigma_{2}I_{\text{m}}\left(t\right)+\sigma g\left(1-e\right)I_{\text{pm}}\left(t\right)-\left(\delta_{2}+\mu \right)R_{\text{m}}\left(t\right),\\ \frac{dR_{\text{pm}}\left(t\right)}{dt}=\sigma \left(1-g\right)\left(1-e\right)I_{\text{pm}}\left(t\right)-\left(\delta_{3}+\mu \right)R_{\text{pm}}\left(t\right). \end{array}\right. \end{array}$$

Initial conditions of system (1) are

(2) $$\begin{array}{c} S\left(0\right)\geq 0,\\ I_{\text{p}}\left(0\right)\geq 0,\\ I_{\text{m}}\left(0\right)\geq 0,\\ I_{\text{pm}}\left(0\right)\geq 0,\\ R_{\text{p}}\left(0\right)\geq 0,\\ R_{\text{m}}\left(0\right)\geq 0,\\ R_{\text{pm}}\left(0\right)\geq 0. \end{array}$$

## 3. Qualitative Analysis

In this section, the qualitative behaviours of the model such as the invariant region, positivity of future solution, equilibrium points and their stability analysis, basic reproduction number, and sensitivity analysis are investigated.

### 3.1. Invariant Region

To get the invariant region in which the solution of the model is bounded, we first consider *N*(*t*)=*S*(*t*)+*I*_p_(*t*)+*I*_m_(*t*)+*I*_pm_(*t*)+*R*_p_(*t*)+*R*_m_(*t*)+*R*_pm_(*t*). Then,

(3) $$\begin{array}{c} \frac{dN\left(t\right)}{dt}=\pi -\mu N\left(t\right)-\alpha_{1}\left(I_{\text{p}}\left(t\right)+I_{\text{pm}}\left(t\right)\right)-\alpha_{2}\left(I_{\text{m}}\left(t\right)+I_{\text{pm}}\left(t\right)\right). \end{array}$$

If *α*_1_=*α*_2_=0, then equation (3) becomes

(4) $$\begin{array}{c} \frac{dN\left(t\right)}{dt}\leq \pi -\mu N\left(t\right). \end{array}$$

After solving equation (4), we get

(5) $$\begin{array}{c} \Omega =\left\{\left(S,I_{\text{p}},I_{\text{m}},I_{\text{pm}},R_{\text{p}},R_{\text{m}},R_{\text{pm}}\right)\in {ℜ}^{7}\text{: }0\leq N\leq \frac{\pi }{\mu }\right\}. \end{array}$$

Therefore, the invariant region of the model becomes *Ω*.

### 3.2. Positivity of the Solution

**Theorem 1 .**

If *S*_0_ > 0, *I*_p_0__ > 0, *I*_m_0__ > 0, *I*_pm_0__ > 0, *R*_p_0__ > 0, *R*_m_0__ > 0, *R*_pm_0__ > 0, then all the solution sets (*S*(*t*), *I*_p_(*t*), *I*_m_(*t*), *I*_pm_(*t*), *R*_p_(*t*), *R*_m_(*t*), *R*_pm_(*t*)) are positive for future time.

**Proof**

First, let us take *t*_1_ as

(6) $$\begin{array}{c} t_{1}=\sup\left\{t>0:S\left(\tau \right)>0,I_{\text{p}}\left(\tau \right)>0,I_{\text{m}}\left(\tau \right)>0,I_{\text{pm}}\left(\tau \right)>0,R_{\text{p}}\left(\tau \right)>0,R_{\text{m}}\left(\tau \right)>0,R_{\text{pm}}\left(\tau \right)>0,\;\text{for all }\tau \in \left[0,t\right]\right\}. \end{array}$$

Consider *S*_0_ ≥ 0, *I*_p_0__ ≥ 0, *I*_m_0__ ≥ 0, *I*_pm_0__ ≥ 0, *R*_p_0__ ≥ 0, *R*_m_0__ ≥ 0, *R*_pm_0__ ≥ 0; thus, *t*_1_ > 0. If *t*_1_ < *∞*, then necessarily *S* or *I*_p_ or *I*_m_ or *I*_pm_ or *R*_p_ or *R*_m_ or *R*_pm_ is equal to zero at *t*_1_. From equation (1),

(7) $$\begin{array}{c} \frac{dS\left(t\right)}{dt}=\pi +\delta_{1}R_{\text{p}}\left(t\right)+\delta_{2}R_{\text{m}}\left(t\right)+\delta_{3}R_{\text{pm}}\left(t\right)-\left(f_{1}+f_{2}+\mu \right)S\left(t\right). \end{array}$$

Using variation formula, equation (7) can be solved at *t*_1_:

(8) $$\begin{array}{c} S\left(t_{1}\right)=S\left(0\right)\exp\left[-\int_{0}^{t_{1}}\left(f_{1}+f_{2}+\mu \right)\left(s\right)ds\right]+\int_{0}^{t_{1}}\left(\pi +\delta_{1}R_{\text{p}}+\delta_{2}R_{\text{m}}+\delta_{3}R_{\text{pm}}\right)\exp\left[-\int_{s}^{t_{1}}\left(f_{1}+f_{2}+\mu \right)\left(\tau \right)d\tau \right]ds. \end{array}$$

Accordingly, all the variables are nonnegative in [0, *t*_1_]; then, *S*(*t*_1_) > 0.

In a similar fashion, we can show *I*_p_(*t*_1_) > 0, *I*_m_(*t*_1_) > 0, *I*_pm_(*t*_1_) > 0, *R*_p_(*t*_1_) > 0, *R*_m_(*t*_1_) > 0, and *R*_pm_(*t*_1_) > 0 which is a contradiction. Hence, *t*_1_=*∞*.

### 3.3. Disease-Free Equilibrium (DFE)

Eliminating *I*_p_(*t*), *I*_m_(*t*), and *I*_pm_(*t*) from equation (1) and solving for *S*(*t*) give DFE:

(9) $$\begin{array}{c} E_{0}=\left(\frac{\pi }{\mu },0,0,0,0,0,0\right). \end{array}$$

### 3.4. Basic Reproduction Number (*ℜ*_0_)

Considering only the infected compartment and applying the next generation matrix give the following eigenvalues:

(10) $$\begin{array}{c} \lambda_{1}^{*}=\frac{a\pi }{\mu \left(\sigma_{1}+\alpha_{1}+\mu \right)}={ℜ}_{\text{0p}},\\ \lambda_{2}^{*}=\frac{b\pi }{\mu \left(\sigma_{2}+\alpha_{2}+\mu \right)}={ℜ}_{\text{0m}},\\ \lambda_{3}^{*}=0. \end{array}$$

Since the basic reproduction number is the dominant eigenvalue of the next generation matrix,

(11) $$\begin{array}{c} {ℜ}_{0}=\max\left\{{ℜ}_{\text{0p}},{ℜ}_{\text{0m}}\right\}. \end{array}$$

### 3.5. Local Stability of Disease-Free Equilibrium

After obtaining the Jacobian matrix of the system at the disease-free equilibrium point, we obtained the following theorem.

**Theorem 2 .**

The disease-free equilibrium point is locally asymptotically stable if *ℜ*_0_ < 1, otherwise unstable.

### 3.6. Global Asymptotic Stability of Disease-Free Equilibrium

To investigate the global stability of disease-free equilibrium, we used the technique implemented in [9]. First, full pneumonia-meningitis model (1) can be re-written as

(12) $$\begin{array}{c} \frac{dX}{dt}=F\left(X,Z\right),\\ \frac{dZ}{dt}=G\left(X,Z\right),\\ G\left(X,0\right)=0, \end{array}$$

where *X* stands for the uninfected population, that is *X*=(*S*, *R*_p_, *R*_m_, *R*_pm_), and *Z* stands for the infected population, that is *Z*=(*I*_p_, *I*_m_, *I*_pm_). The disease-free equilibrium point of the model is denoted by *U*=(*X*^*∗*^, 0).

For the point *U*=(*X*^*∗*^, 0) to be globally asymptotically stable equilibrium for the model provided that *ℜ*_0_ < 1 (which is locally asymptotically stable) and the following conditions must be met:

 
(*H*_1_): for (*dX*/*dt*)=*F*(*X*, 0), *X*^*∗*^ is globally asymptotically stable.
 
(*H*_2_): $G\left(X,Z\right)=AZ-\tilde{G}\left(X,Z\right),\tilde{G}\left(X,Z\right)\geq 0$ for (*X*, *Z*) ∈ *Ω*

If model (1) met the aforementioned two criteria, then the following theorem holds.

**Theorem 3 .**

The point *U*=(*X*^*∗*^, 0) is globally asymptotically stable equilibrium provided that *ℜ*_0_ < 1 and the conditions (*H*_1_) and (*H*_2_) are satisfied.

**Proof**

From system (1), we can get *F*(*X*, *Z*) and *G*(*X*, *Z*):

(13) $$\begin{array}{c} F\left(X,Z\right)=\left(\begin{array}{c} \pi +\delta_{1}R_{\text{p}}+\delta_{2}R_{\text{m}}+\delta_{3}R_{\text{pm}}-\left(f_{1}+f_{2}+\mu \right)S\\ \sigma_{1}I_{\text{p}}+\sigma eI_{\text{pm}}-\left(\delta_{1}+\mu \right)R_{\text{p}}\\ \sigma_{2}I_{\text{m}}+\sigma g\left(1-e\right)I_{\text{pm}}-\left(\delta_{2}+\mu \right)R_{\text{m}}\\ \sigma \left(1-g\right)\left(1-e\right)I_{\text{pm}}-\left(\delta_{3}+\mu \right)R_{\text{pm}} \end{array}\right),\\ G\left(X,Z\right)=\left(\begin{array}{c} f_{1}S-\left(f_{2}+\sigma_{1}+\alpha_{1}+\mu \right)I_{\text{p}}\\ f_{2}S-\left(f_{1}+\sigma_{2}+\alpha_{2}+\mu \right)I_{\text{m}}\\ f_{2}I_{\text{p}}+f_{1}I_{\text{m}}-\left(\sigma +\alpha_{1}+\alpha_{2}+\mu \right)I_{\text{pm}} \end{array}\right). \end{array}$$

Consider the reduced system:

(14) $$\begin{array}{c} {\left.\frac{dX}{dt}\right|}_{Z=0}=\left(\begin{array}{c} \pi -\mu S\\ 0\\ 0\\ 0 \end{array}\right). \end{array}$$

From equation (14), it is obvious that *X*^*∗*^=((*π*/*μ*), 0) is the global asymptotic point. This can be verified from the solution, namely, *S*=(*π*/*μ*)+(*S*(0) − (*π*/*μ*))*e*^−*μt*^. As *t*⟶*∞*, the solution (*S*)⟶(*π*/*μ*), implying the global convergence of (14) in *Ω*.

Let

(15) $$\begin{array}{c} A=\left(\begin{array}{ccc} a-\left({\text{sigma}}_{1}+\alpha_{1}+\mu \right) & 0 & a\\ 0 & -\left(\sigma_{2}+\alpha_{2}+\mu \right) & 0\\ 0 & 0 & -\left(\sigma +\alpha_{1}+\alpha_{2}+\mu \right) \end{array}\right). \end{array}$$

Then, *G*(*X*, *Z*) can be written as $G\left(X,Z\right)=AZ-\tilde{G}\left(X,Z\right)$, where

(16) $$\begin{array}{c} \tilde{G}\left(X,Z\right)=\left(\begin{array}{c} {\tilde{G}}_{1}\left(X,Z\right)\\ {\tilde{G}}_{2}\left(X,Z\right)\\ {\tilde{G}}_{3}\left(X,Z\right) \end{array}\right)=\left(\begin{array}{c} a\left(I_{\text{m}}+I_{\text{pm}}\right)\left(1-\frac{S}{N}\right)+f_{2}I_{\text{p}}\\ f_{1}I_{\text{m}}\\ -\left(f_{2}I_{\text{p}}+f_{1}I_{\text{m}}\right) \end{array}\right), \end{array}$$

where ${\tilde{G}}_{2}\left(X,Z\right)<0$ which leads to $\tilde{G}\left(X,Z\right)<0$, which means the second condition (*H*_2_) is not satisfied, so *U*=(*X*^*∗*^, 0) may not be globally asymptotically stable when *ℜ*_0_ < 1.

### 3.7. Sensitivity Analysis

Here we performed sensitivity analysis in order to check the effect of each parameter in the expansion as well in controlling pneumonia and meningitis infection as well as their coinfection. To perform sensitivity analysis, we used a method outlined in [7]. Sensitivity index of *ℜ*_0_ with respect to parameter, say *y*, is given by Λ_*y*_^*ℜ*0^=(∂*ℜ*_0_/∂*y*)(*y*/*ℜ*_0_). Since *ℜ*_0_=max{*ℜ*_0p_, *ℜ*_0m_}, we obtained sensitivity analysis of *ℜ*_0p_ and *ℜ*_0m_ separately in the following way:

(17) $$\begin{array}{c} \Lambda_{a}^{{ℜ}_{\text{0p}}}=\frac{\partial {ℜ}_{\text{0p}}}{\partial a}\frac{a}{{ℜ}_{\text{0p}}}=\frac{\pi }{\mu \left(\sigma_{1}+\alpha_{1}+\mu \right)}\frac{a\mu \left(\sigma_{1}+\alpha_{1}+\mu \right)}{a\pi }=1>0,\\ \Lambda_{\alpha_{1}}^{{ℜ}_{\text{0p}}}=\frac{\partial {ℜ}_{\text{0p}}}{\partial \alpha_{1}}\frac{\alpha_{1}}{{ℜ}_{\text{0p}}}=-\frac{\alpha_{1}}{\left(\sigma_{1}+\alpha_{1}+\mu \right)}<0,\\ \Lambda_{\sigma_{1}}^{{ℜ}_{\text{0p}}}=\frac{\partial {ℜ}_{\text{0p}}}{\partial \sigma_{1}}\frac{\sigma_{1}}{{ℜ}_{\text{0p}}}=-\frac{\sigma_{1}}{\left(\sigma_{1}+\alpha_{1}+\mu \right)}<0,\\ \Lambda_{\mu }^{{ℜ}_{\text{0p}}}=\frac{\partial {ℜ}_{\text{0p}}}{\partial \mu }\frac{\mu }{{ℜ}_{\text{0p}}}=-\frac{\left(\sigma_{1}+\alpha_{1}+2\mu \right)}{\left(\sigma_{1}+\alpha_{1}+\mu \right)}<0,\\ \Lambda_{b}^{{ℜ}_{\text{0m}}}=\frac{\partial {ℜ}_{\text{0m}}}{\partial b}\frac{b}{{ℜ}_{\text{0m}}}=\frac{\pi }{\mu \left(\sigma_{2}+\alpha_{2}+\mu \right)}\frac{b\mu \left(\sigma_{2}+\alpha_{2}+\mu \right)}{b\pi }=1>0,\\ \Lambda_{\alpha_{2}}^{{ℜ}_{\text{0m}}}=\frac{\partial {ℜ}_{\text{0m}}}{\partial \alpha_{2}}\frac{\alpha_{2}}{{ℜ}_{\text{0m}}}=-\frac{\alpha_{2}}{\left(\sigma_{2}+\alpha_{2}+\mu \right)}<0,\\ \Lambda_{\sigma_{2}}^{{ℜ}_{02}}=\frac{\partial {ℜ}_{\text{0m}}}{\partial \sigma_{2}}\frac{\sigma_{2}}{{ℜ}_{\text{0m}}}=-\frac{\sigma_{2}}{\left(\sigma_{2}+\alpha_{2}+\mu \right)}<0,\\ \Lambda_{\mu }^{{ℜ}_{\text{0m}}}=\frac{\partial {ℜ}_{\text{0m}}}{\partial \mu }\frac{\mu }{{ℜ}_{\text{0m}}}=-\frac{\left(\sigma_{2}+\alpha_{2}+2\mu \right)}{\left(\sigma_{2}+\alpha_{2}+\mu \right)}<0. \end{array}$$

The above computation shows that pneumonia, meningitis, and their coinfection will be expanded if these parameters have a positive index, which is increased by keeping the other parameters constant. However, those parameters whose indices are negative have a great role in decreasing the diseases if their values are increased by keeping other parameters constant. From this result, we took prevention and treatment for both diseases to be considered in optimal control analysis in the next section.

## 4. Optimal Control Analysis

In this section, we extended the basic model in equation (1) to optimal control by incorporating five controls which have a significant effect in controlling the expansion of coepidemics of pneumonia and meningitis. The interventions are as follows:

1. *u*_1_: pneumonia prevention effort
2. *u*_2_: meningitis prevention effort
3. *u*_3_: pneumonia treating effort
4. *u*_4_: meningitis treating effort

After incorporating the above controls, the extended model becomes

(18) $$\begin{array}{c} \left\{\begin{array}{c} \frac{dS\left(t\right)}{dt}=\pi +\delta_{1}R_{\text{p}}\left(t\right)+\delta_{2}R_{\text{m}}\left(t\right)+\delta_{3}R_{\text{pm}}\left(t\right)-\left(\left(1-u_{1}\right)f_{1}+\left(1-u_{2}\right)f_{2}+\mu \right)S\left(t\right),\\ \frac{dI_{\text{p}}\left(t\right)}{dt}=\left(1-u_{1}\right)f_{1}S\left(t\right)-\left(1-u_{2}\right)f_{2}I_{\text{p}}\left(t\right)-\left(\sigma_{1}+u_{3}\right)I_{\text{p}}\left(t\right)-\left(\alpha_{1}+\mu \right)I_{\text{p}}\left(t\right),\\ \frac{dI_{\text{m}}\left(t\right)}{dt}=\left(1-u_{2}\right)f_{2}S\left(t\right)-\left(1-u_{1}\right)f_{1}I_{\text{m}}\left(t\right)-\left(\sigma_{2}+u_{4}\right)I_{\text{m}}\left(t\right)-\left(\alpha_{2}+\mu \right)I_{\text{m}}\left(t\right),\\ \frac{dI_{\text{pm}}\left(t\right)}{dt}=\left(1-u_{2}\right)f_{2}I_{\text{p}}\left(t\right)+\left(1-u_{1}\right)f_{1}I_{\text{m}}\left(t\right)-\left(\sigma +u_{3}+u_{4}\right)I_{\text{pm}}\left(t\right)-\left(\alpha_{1}+\alpha_{2}+\mu \right)I_{\text{pm}}\left(t\right),\\ \frac{dR_{\text{p}}\left(t\right)}{dt}=\left(\sigma_{1}+u_{3}\right)I_{\text{p}}\left(t\right)+\left(\sigma e+u_{3}\right)I_{\text{pm}}\left(t\right)-\left(\delta_{1}+\mu \right)R_{\text{p}}\left(t\right),\\ \frac{dR_{\text{m}}\left(t\right)}{dt}=\left(\sigma_{2}+u_{4}\right)I_{\text{m}}\left(t\right)+\left(\sigma g\left(1-e\right)+u_{4}\right)I_{\text{pm}}\left(t\right)-\left(\delta_{2}+\mu \right)R_{\text{m}}\left(t\right),\\ \frac{dR_{\text{pm}}\left(t\right)}{dt}=\left(\sigma \left(1-g\right)\left(1-e\right)+u_{3}+u_{4}\right)I_{\text{pm}}\left(t\right)-\left(\delta_{3}+\mu \right)R_{\text{pm}}\left(t\right). \end{array}\right. \end{array}$$

Being Lebesgue measurable of *U* is crucial for studying the optimal levels of *U*={(*u*_1_(*t*), *u*_2_(*t*), *u*_3_(*t*), *u*_4_(*t*)) : 0 ≤ *u*_1_ < 1,0 ≤ *u*_2_ < 1,0 ≤ *u*_3_ < 1,0 ≤ *u*_4_ < 1,0 ≤ *t* ≤ *T*}. The main target is to get *U* and *I*_p_(*t*), *I*_m_(*t*), and *I*_pm_(*t*), which minimize the objective function *J*, given by

(19) $$\begin{array}{c} J=\underset{u_{1},u_{2},u_{3},u_{4}}{\min}\int_{0}^{t_{f}}\left(c_{1}I_{\text{p}}\left(t\right)+c_{2}I_{\text{m}}\left(t\right)+c_{3}I_{\text{pm}}\left(t\right)+\frac{1}{2}\overset{4}{\underset{i=1}{\sum }}w_{i}u_{i}^{2}\right)dt, \end{array}$$

where *c*_1_, *c*_2_, *c*_3_, and *w*_*i*_ are positive. The expression (1/2)*w*_*i*_*u*_*i*_^2^ represents costs. Our aim is to minimize infectious compartments and costs. Therefore, we want to get optimal controls (*u*_1_^*∗*^, *u*_2_^*∗*^, *u*_3_^*∗*^, *u*_4_^*∗*^) in which

(20) $$\begin{array}{c} J\left(u_{1}^{*},u_{2}^{*},u_{3}^{*},u_{4}^{*}\right)=\min\left\{\left(J\left(u_{1},u_{2},u_{3},u_{4}\right)/u_{i}\right)\in U\right\}. \end{array}$$

### 4.1. The Hamiltonian and Optimality System

Here, Hamiltonian (*H*) is derived by applying Pontryagin's maximum principle in the same way as described in [11], which is defined as

(21) $$\begin{array}{c} H\left(S\left(t\right),I_{\text{p}}\left(t\right),I_{\text{m}}\left(t\right),I_{\text{pm}}\left(t\right),R_{\text{p}}\left(t\right),R_{\text{m}}\left(t\right),R_{\text{pm}}\left(t\right)\right)=L\left(I_{\text{p}}\left(t\right),I_{\text{m}}\left(t\right),I_{\text{pm}}\left(t\right),u_{1},u_{2},u_{3},u_{4},t\right)+\lambda_{1}\frac{ds\left(t\right)}{dt}+\lambda_{2}\left(t\right)\frac{dI_{\text{p}}\left(t\right)}{dt}+\lambda_{3}\left(t\right)\frac{dI_{\text{m}}\left(t\right)}{dt}+\lambda_{4}\left(t\right)\frac{dI_{\text{pm}}\left(t\right)}{dt}+\lambda_{5}\left(t\right)\frac{dR_{\text{p}}\left(t\right)}{dt}+\lambda_{6}\left(t\right)\frac{dR_{\text{m}}\left(t\right)}{dt}+\lambda_{7}\left(t\right)\frac{dR_{\text{pm}}\left(t\right)}{dt}, \end{array}$$

where *L*(*I*_p_(*t*), *I*_m_(*t*), *I*_pm_(*t*), *u*_1_, *u*_2_, *u*_3_, *u*_4_, *t*)=*c*_1_*I*_p_(*t*)+*c*_2_*I*_m_(*t*)+*c*_3_*I*_pm_(*t*)+1/2∑_*i*=1_^4^*w*_*i*_*u*_*i*_^2^, *λ*_*i*_, *i*=1,2,3,4,5,6,7 are the adjoint variable functions.

**Theorem 4 .**

For an optimal control set *u*_1_, *u*_2_, *u*_3_, *u*_4_ that minimizes *J* over *U*, there are adjoint variables, *λ*_1_,…, *λ*_7_ such that

(22) $$\begin{array}{c} \frac{d\lambda_{1}}{dt}=\lambda_{1}\left(\left(1-u_{1}\right)f_{1}+\left(1-u_{2}\right)f_{2}+\mu \right)-\lambda_{2}\left(1-u_{1}\right)f_{1}-\lambda_{3}\left(1-u_{2}\right)f_{2},\\ \frac{d\lambda_{2}}{dt}=-c_{1}-\lambda_{1}\left(1-u_{1}\right)\frac{aS}{N}-\lambda_{2}\left(\left(1-u_{1}\right)\frac{aS}{N}+\left(1-u_{2}\right)f_{2}+\left(\sigma_{1}+u_{3}+\alpha_{1}+\mu \right)\right)-\lambda_{4}\left(\left(1-u_{2}\right)f_{2}+\left(1-u_{1}\right)\frac{aI_{\text{m}}}{N}\right)-\lambda_{5}\left(\sigma_{1}+u_{3}\right),\\ \frac{d\lambda_{3}}{dt}=-c_{2}+\lambda_{1}\left(1-u_{2}\right)\frac{bS}{N}+\lambda_{2}\left(1-u_{2}\right)\frac{bI_{\text{p}}}{N}-\lambda_{3}\left(\sigma_{2}+\alpha_{2}+u_{4}+\left(1-u_{1}\right)f_{1}+\left(1-u_{1}\right)f_{1}-\left(1-u_{2}\right)\frac{bS}{N}\right)-\lambda_{4}\left(\left(1-u_{2}\right)\frac{bI_{\text{p}}}{N}+\left(1-u_{1}\right)f_{1}\right)-\lambda_{6}\left(\sigma_{2}+u_{4}\right),\\ \frac{d\lambda_{4}}{dt}=-c_{3}+\lambda_{1}\left(\left(1-u_{1}\right)\frac{aS}{N}+\left(1-u_{2}\right)\frac{bS}{N}\right)-\lambda_{2}\left(\left(1-u_{2}\right)\frac{bI_{\text{p}}}{N}-\left(1-u_{1}\right)\frac{aS}{N}\right)-\lambda_{3}\left(\left(1-u_{1}\right)\frac{aI_{\text{m}}}{N}-\left(1-u_{2}\right)\frac{bS}{N}\right)-\lambda_{4}\left(\sigma +\alpha_{1}+\alpha_{2}+\mu +u_{3}+u_{4}-\left(1-u_{2}\right)\frac{bI_{\text{p}}}{N}-\left(1-u_{1}\right)\frac{aI_{\text{m}}}{N}\right)-\lambda_{5}\left(\sigma e+u_{3}\right)-\lambda_{6}\left(\sigma g\left(1-e\right)+u_{4}\right)-\lambda_{7}\left(\sigma \left(1-g\right)\left(1-e\right)+u_{3}+u_{4}\right),\\ \frac{d\lambda_{5}}{dt}=\lambda_{5}\left(\delta_{1}+\mu \right)-\lambda_{1}\delta_{1},\\ \frac{d\lambda_{6}}{dt}=\lambda_{6}\left(\delta_{2}+\mu \right)-\lambda_{1}\delta_{2},\\ \frac{d\lambda_{7}}{dt}=\lambda_{7}\left(\delta_{3}+\mu \right)-\lambda_{1}\delta_{3}, \end{array}$$

with the condition *λ*_*i*_(*t*_*f*_)=0, *i*=1,…, 7.

The characterized control sets are:

(23) $$\begin{array}{c} u_{1}^{*}\left(t\right)=\max\left\{0,\min\left(1,\Phi_{1}\right)\right\},\\ u_{2}^{*}\left(t\right)=\max\left\{0,\min\left(1,\Phi_{2}\right)\right\},\\ u_{3}^{*}\left(t\right)=\max\left\{0,\min\left(1,\Phi_{3}\right)\right\},\\ u_{4}^{*}\left(t\right)=\max\left\{0,\min\left(1,\Phi_{4}\right)\right\}, \end{array}$$

where

(24) $$\begin{array}{c} \Phi_{1}=\frac{f_{1}S\left(\lambda_{2}-\lambda_{1}\right)+f_{1}I_{\text{p}}\left(\lambda_{4}-\lambda_{3}\right)}{w_{1}},\\ \Phi_{2}=\frac{f_{2}S\left(\lambda_{3}-\lambda_{1}\right)+f_{2}I_{\text{p}}\left(\lambda_{4}-\lambda_{2}\right)}{w_{2}},\\ \Phi_{3}=\frac{\lambda_{2}I_{\text{p}}+I_{\text{pm}}\left(\lambda_{4}-\lambda_{7}\right)-\lambda_{5}\left(I_{\text{p}}+I_{\text{pm}}\right)}{w_{3}},\\ \Phi_{4}=\frac{\lambda_{3}I_{\text{m}}+I_{\text{pm}}\left(\lambda_{4}-\lambda_{7}\right)-\lambda_{6}\left(I_{\text{m}}+I_{\text{pm}}\right)}{w_{4}}. \end{array}$$

**Proof**

Applying Pontryagin's maximum principle gives the adjoint systems:

(25) $$\begin{array}{c} \frac{d\lambda_{1}}{dt}=-\frac{dH}{dS}=\lambda_{1}\left(\left(1-u_{1}\right)f_{1}+\left(1-u_{2}\right)f_{2}+\mu \right)-\lambda_{2}\left(1-u_{1}\right)f_{1}-\lambda_{3}\left(1-u_{2}\right)f_{2},\\ \frac{d\lambda_{2}}{dt}=-\frac{dH}{dI_{\text{p}}}=-c_{1}-\lambda_{1}\left(1-u_{1}\right)\frac{aS}{N}-\lambda_{2}\left(\left(1-u_{1}\right)\frac{aS}{N}+\left(1-u_{2}\right)f_{2}+\left(\sigma_{1}+u_{3}+\alpha_{1}+\mu \right)\right)-\lambda_{4}\left(\left(1-u_{2}\right)f_{2}+\left(1-u_{1}\right)\frac{aI_{\text{m}}}{N}\right)-\lambda_{5}\left(\sigma_{1}+u_{3}\right),\\ \frac{d\lambda_{3}}{dt}=-\frac{dH}{dI_{\text{m}}}=-c_{2}+\lambda_{1}\left(1-u_{2}\right)\frac{bS}{N}+\lambda_{2}\left(1-u_{2}\right)\frac{bI_{\text{p}}}{N}-\lambda_{3}\left(\sigma_{2}+\alpha_{2}+u_{4}+\left(1-u_{1}\right)f_{1}-\left(1-u_{2}\right)\frac{bS}{N}\right)-\lambda_{4}\left(\left(1-u_{2}\right)\frac{bI_{\text{p}}}{N}+\left(1-u_{1}\right)f_{1}\right)-\lambda_{6}\left(\sigma_{2}+u_{4}\right),\\ \frac{d\lambda_{4}}{dt}=-\frac{dH}{dI_{\text{pm}}}=-c_{3}+\lambda_{1}\left(\left(1-u_{1}\right)\frac{aS}{N}+\left(1-u_{2}\right)\frac{bS}{N}\right)-\lambda_{2}\left(\left(1-u_{2}\right)\frac{bI_{\text{p}}}{N}-\left(1-u_{1}\right)\frac{aS}{N}\right)-\lambda_{3}\left(\left(1-u_{1}\right)\frac{aI_{\text{m}}}{N}-\left(1-u_{2}\right)\frac{bS}{N}\right)-\lambda_{4}\left(\sigma +\alpha_{1}+\alpha_{2}+\mu +u_{3}+u_{4}-\left(1-u_{2}\right)\frac{bI_{\text{p}}}{N}-\left(1-u_{1}\right)\frac{aI_{\text{m}}}{N}\right)-\lambda_{5}\left(\sigma e+u_{3}\right)-\lambda_{6}\left(\sigma g\left(1-e\right)+u_{4}\right)-\lambda_{7}\left(\sigma \left(1-g\right)\left(1-e\right)+u_{3}+u_{4}\right),\\ \frac{d\lambda_{5}}{dt}=-\frac{dH}{dR_{\text{p}}}=\lambda_{5}\left(\delta_{1}+\mu \right)-\lambda_{1}\delta_{1},\\ \frac{d\lambda_{6}}{dt}=-\frac{dH}{dR_{\text{m}}}=\lambda_{6}\left(\delta_{2}+\mu \right)-\lambda_{1}\delta_{2},\\ \frac{d\lambda_{7}}{dt}=-\frac{dH}{dR_{\text{pm}}}=\lambda_{7}\left(\delta_{3}+\mu \right)-\lambda_{1}\delta_{3}. \end{array}$$

Now to obtain the time varying controls, we used the equation, (∂*H*/∂*u*_*i*_)=0  at  *u*_*i*_^*∗*^, for *i*=1,…, 4 and obtained the following:

(26) $$\begin{array}{c} u_{1}^{*}=\frac{f_{1}S\left(\lambda_{2}-\lambda_{1}\right)+f_{1}I_{\text{p}}\left(\lambda_{4}-\lambda_{3}\right)}{w_{1}},\\ u_{2}^{*}=\frac{f_{2}S\left(\lambda_{3}-\lambda_{1}\right)+f_{2}I_{\text{p}}\left(\lambda_{4}-\lambda_{2}\right)}{w_{2}},\\ u_{3}^{*}=\frac{\lambda_{2}I_{\text{p}}+I_{\text{pm}}\left(\lambda_{4}-\lambda_{7}\right)-\lambda_{5}\left(I_{\text{p}}+I_{\text{pm}}\right)}{w_{3}},\\ u_{4}^{*}=\frac{\lambda_{3}I_{\text{m}}+I_{\text{pm}}\left(\lambda_{4}-\lambda_{7}\right)-\lambda_{6}\left(I_{\text{m}}+I_{\text{pm}}\right)}{w_{4}}. \end{array}$$

The controls can be written as

(27) $$\begin{array}{c} u_{1}^{*}=\left\{\begin{array}{cc} \Phi_{1}, & \text{if }0<\Phi_{1}<1,\\ 0, & \text{if }\Phi_{1}\leq 0,\\ 1, & \text{if }\Phi_{1}\geq 1, \end{array}\right.\\ u_{2}^{*}=\left\{\begin{array}{cc} \Phi_{2}, & \text{if }0<\Phi_{2}<1,\\ 0, & \text{if }\Phi_{2}\leq 0,\\ 1, & \text{if }\Phi_{2}\geq 1, \end{array}\right.\\ u_{3}^{*}=\left\{\begin{array}{cc} \Phi_{3}, & \text{if }0<\Phi_{3}<1,\\ 0, & \text{if }\Phi_{3}\leq 0,\\ 1, & \text{if }\Phi_{3}\geq 1, \end{array}\right.\\ u_{4}^{*}=\left\{\begin{array}{cc} \Phi_{4}, & \text{if }0<\Phi_{4}<1,\\ 0, & \text{if }\Phi_{4}\leq 0,\\ 1, & \text{if }\Phi_{4}\geq 1, \end{array}\right.\\ u_{5}^{*}=\left\{\begin{array}{cc} \Phi_{5}, & \text{if }0<\Phi_{5}<1,\\ 0, & \text{if }\Phi_{5}\leq 0,\\ 1, & \text{if }\Phi_{5}\geq 1. \end{array}\right. \end{array}$$

The compact representation of the controls:

(28) $$\begin{array}{c} u_{1}^{*}\left(t\right)=\max\left\{0,\min\left(1,\Phi_{1}\right)\right\},\\ u_{2}^{*}\left(t\right)=\max\left\{0,\min\left(1,\Phi_{2}\right)\right\},\\ u_{3}^{*}\left(t\right)=\max\left\{0,\min\left(1,\Phi_{3}\right)\right\},\\ u_{4}^{*}\left(t\right)=\max\left\{0,\min\left(1,\Phi_{4}\right)\right\},\\ u_{5}^{*}\left(t\right)=\max\left\{0,\min\left(1,\Phi_{5}\right)\right\},\\ \Phi_{1}=\frac{f_{1}S\left(\lambda_{2}-\lambda_{1}\right)+f_{1}I_{\text{p}}\left(\lambda_{4}-\lambda_{3}\right)}{w_{1}},\\ \Phi_{2}=\frac{f_{2}S\left(\lambda_{3}-\lambda_{1}\right)+f_{2}I_{\text{p}}\left(\lambda_{4}-\lambda_{2}\right)}{w_{2}},\\ \Phi_{3}=\frac{\lambda_{2}I_{\text{p}}+I_{\text{pm}}\left(\lambda_{4}-\lambda_{7}\right)-\lambda_{5}\left(I_{\text{p}}+I_{\text{pm}}\right)}{w_{3}},\\ \Phi_{4}=\frac{\lambda_{3}I_{\text{m}}+I_{\text{pm}}\left(\lambda_{4}-\lambda_{7}\right)-\lambda_{6}\left(I_{\text{m}}+I_{\text{pm}}\right)}{w_{4}}. \end{array}$$

Then, the obtained optimality system is

(29) $$\begin{array}{c} \frac{dS}{dt}=\pi +\delta_{1}R_{\text{p}}+\delta_{2}R_{\text{m}}+\delta_{3}R_{\text{pm}}-\left(\left(1-u_{1}\right)f_{1}+\left(1-u_{2}\right)f_{2}+\mu \right)S,\\ \frac{dI_{\text{m}}}{dt}=\left(1-u_{2}\right)f_{2}S-\left(1-u_{1}\right)f_{1}I_{\text{m}}-\left(\sigma_{2}+u_{4}\right)I_{\text{m}}-\left(\alpha_{2}+\mu \right)I_{\text{m}},\\ \frac{dI_{\text{pm}}}{dt}=\left(1-u_{2}\right)f_{2}I_{\text{p}}+\left(1-u_{1}\right)f_{1}I_{\text{m}}-\left(\sigma +u_{3}+u_{4}\right)I_{\text{pm}}-\left(\alpha_{1}+\alpha_{2}+\mu \right)I_{\text{pm}},\\ \frac{dR_{\text{p}}}{dt}=\left(\sigma_{1}+u_{3}\right)I_{\text{p}}+\left(\sigma e+u_{3}\right)I_{\text{pm}}-\left(\delta_{1}+\mu \right)R_{\text{p}},\\ \frac{dR_{\text{m}}}{dt}=\left(\sigma_{2}+u_{4}\right)I_{\text{m}}+\left(\sigma g\left(1-e\right)+u_{4}\right)I_{\text{pm}}-\left(\delta_{2}+\mu \right)R_{\text{m}},\\ \frac{dR_{\text{pm}}}{dt}=\left(\sigma \left(1-g\right)\left(1-e\right)+u_{3}+u_{4}\right)I_{\text{pm}}-\left(\delta_{3}+\mu \right)R_{\text{pm}},\\ \frac{d\lambda_{1}}{dt}=\lambda_{1}\left(\left(1-u_{1}\right)f_{1}+\left(1-u_{2}\right)f_{2}+\mu \right)-\lambda_{2}\left(1-u_{1}\right)f_{1}-\lambda_{3}\left(1-u_{2}\right)f_{2},\\ \frac{d\lambda_{2}}{dt}=-c_{1}-\lambda_{1}\left(1-u_{1}\right)\frac{aS}{N}-\lambda_{2}\left(\left(1-u_{1}\right)\frac{aS}{N}+\left(1-u_{2}\right)f_{2}+\left(\sigma_{1}+u_{3}+\alpha_{1}+\mu \right)\right)-\lambda_{4}\left(\left(1-u_{2}\right)f_{2}+\left(1-u_{1}\right)\frac{aI_{\text{m}}}{N}\right)-\lambda_{5}\left(\sigma_{1}+u_{3}\right),\\ \frac{d\lambda_{3}}{dt}=-c_{2}+\lambda_{1}\left(1-u_{2}\right)\frac{bS}{N}+\lambda_{2}\left(1-u_{2}\right)\frac{bI_{\text{p}}}{N}-\lambda_{3}\left(\sigma_{2}+\alpha_{2}+u_{4}+\left(1-u_{1}\right)f_{1}-\left(1-u_{2}\right)\frac{bS}{N}\right)-\lambda_{4}\left(\left(1-u_{2}\right)\frac{bI_{\text{p}}}{N}+\left(1-u_{1}\right)f_{1}\right)-\lambda_{6}\left(\sigma_{2}+u_{4}\right),\\ \frac{d\lambda_{4}}{dt}=-c_{3}+\lambda_{1}\left(\left(1-u_{1}\right)\frac{aS}{N}+\left(1-u_{2}\right)\frac{bS}{N}\right)-\lambda_{2}\left(\left(1-u_{2}\right)\frac{bI_{\text{p}}}{N}-\left(1-u_{1}\right)\frac{aS}{N}\right)-\lambda_{3}\left(\left(1-u_{1}\right)\frac{aI_{\text{m}}}{N}-\left(1-u_{2}\right)\frac{bS}{N}\right)-\lambda_{4}\left(\sigma +\alpha_{1}+\alpha_{2}+\mu +u_{3}+u_{4}-\left(1-u_{2}\right)\frac{bI_{\text{p}}}{N}-\left(1-u_{1}\right)\frac{aI_{\text{m}}}{N}\right)-\lambda_{5}\left(\sigma e+u_{3}\right)-\lambda_{6}\left(\sigma g\left(1-e\right)+u_{4}\right)-\lambda_{7}\left(\sigma \left(1-g\right)\left(1-e\right)+u_{3}+u_{4}\right),\\ \frac{d\lambda_{5}}{dt}=\lambda_{5}\left(\delta_{1}+\mu \right)-\lambda_{1}\delta_{1},\\ \frac{d\lambda_{6}}{dt}=\lambda_{6}\left(\delta_{2}+\mu \right)-\lambda_{1}\delta_{2},\\ \frac{d\lambda_{7}}{dt}=\lambda_{7}\left(\delta_{3}+\mu \right)-\lambda_{1}\delta_{3}. \end{array}$$

(30) $$\begin{array}{c} \lambda_{i}\left(t_{f}\right)=0,\;i=1,\ldots ,7,\\ S\left(0\right)=S_{0},\\ I_{\text{p}}\left(0\right)=I_{{\text{p}}_{\text{0}}},\\ I_{\text{m}}\left(0\right)=I_{{\text{m}}_{\text{0}}},\\ I_{\text{pm}}\left(0\right)=I_{{\text{pm}}_{\text{0}}},\\ R_{\text{p}}\left(0\right)=R_{{\text{p}}_{\text{0}}},\\ R_{\text{m}}\left(0\right)=R_{{\text{m}}_{\text{0}}},\\ R_{\text{pm}}\left(0\right)=R_{{\text{pm}}_{\text{0}}}. \end{array}$$

## 5. Numerical Simulation

In this section, we performed numerical simulation of the optimality system. To simulate the system, we used the forward fourth-order Runge–Kutta method to solve the state system and the backward fourth-order Runge–Kutta method for solving the costate system. We have used Maple 18, for simulation.

We proposed the following five strategies for numerical simulation of the optimality system:

- Using prevention effort for both diseases (*u*_1_ ≠ 0, *u*_2_ ≠ 0, *u*_3_=0, *u*_4_=0)
- Prevention effort for pneumonia disease and treatment effort for meningitis disease (*u*_1_ ≠ 0, *u*_4_ ≠ 0, *u*_2_=*u*_3_=0)
- Using prevention effort for meningitis disease and treatment effort for pneumonia disease (*u*_2_ ≠ 0, *u*_3_ ≠ 0, *u*_1_=0, *u*_4_=0)
- Using treatment effort for both diseases (*u*_3_ ≠ 0, *u*_4_ ≠ 0, *u*_1_=*u*_2_=0)
- Using all the intervention efforts (*u*_1_ ≠ 0, *u*_2_ ≠ 0, *u*_3_ ≠ 0, *u*_4_ ≠ 0)

For simulation, we used parameter values listed in Table 1, and we assumed *c*_1_=35, *c*_2_=45, *c*_3_=26, *w*_1_=4, *w*_2_=3, *w*_3_=5, and *w*_4_=6 for simulation. Initial conditions that are used are *S*(0)=1500, *I*_p_(0)=456, *I*_m_(0)=564, *I*_pm_(0)=250, *R*_p_(0)=123, *R*_m_(0)=248, and *R*_pm_(0)=346.

### 5.1. Control with Prevention for Both Diseases

**Figure 2:**
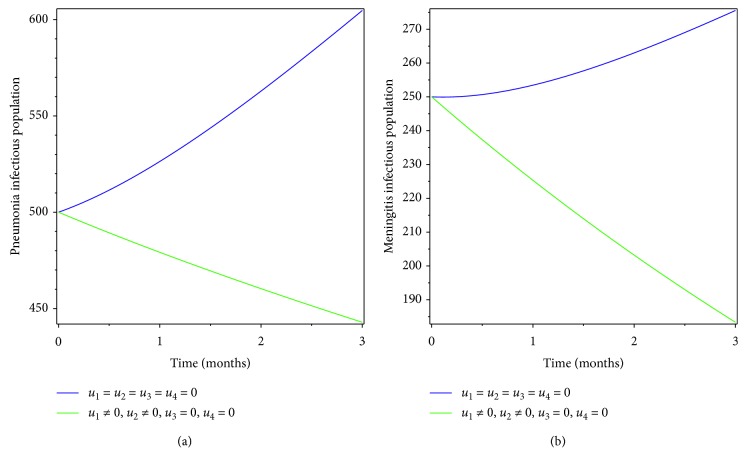
Simulations of optimality system with prevention only.

**Figure 3:**
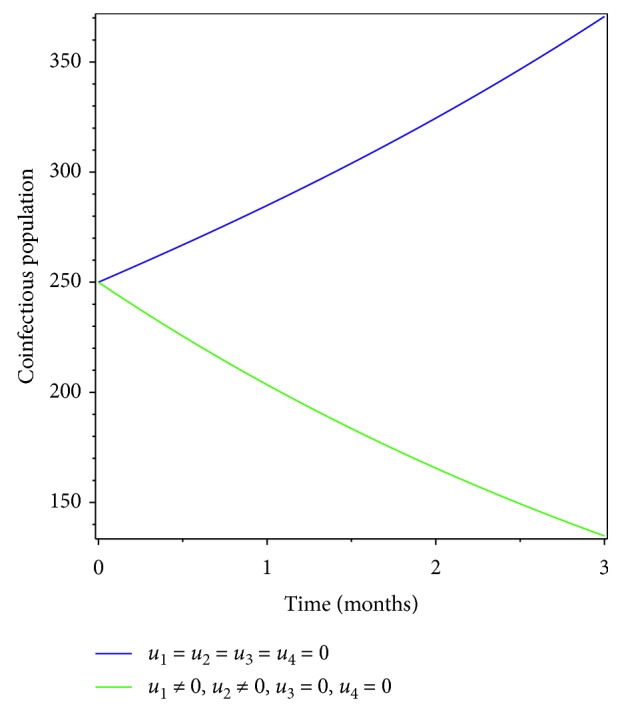
Effect of prevention on coinfectious populations.

Here we applied prevention of both pneumonia and meningitis diseases as the intervention strategy. From the simulation results of Figures 2 and 3, we see that prevention has a great impact in controlling pneumonia-only and meningitis-only infectious population and also in eradicating coinfection of pneumonia and meningitis diseases in the specified time.

### 5.2. Control with Prevention Effort for Pneumonia and Treatment Effort for Meningitis

**Figure 4:**
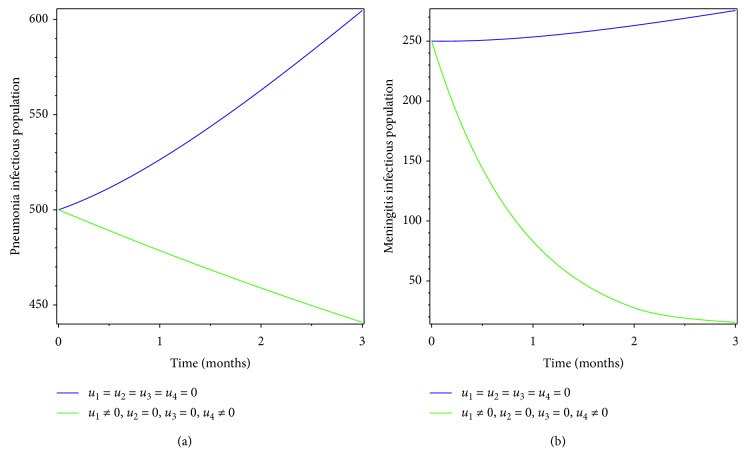
Simulations of optimality system with prevention of pneumonia and treatment of meningitis.

**Figure 5:**
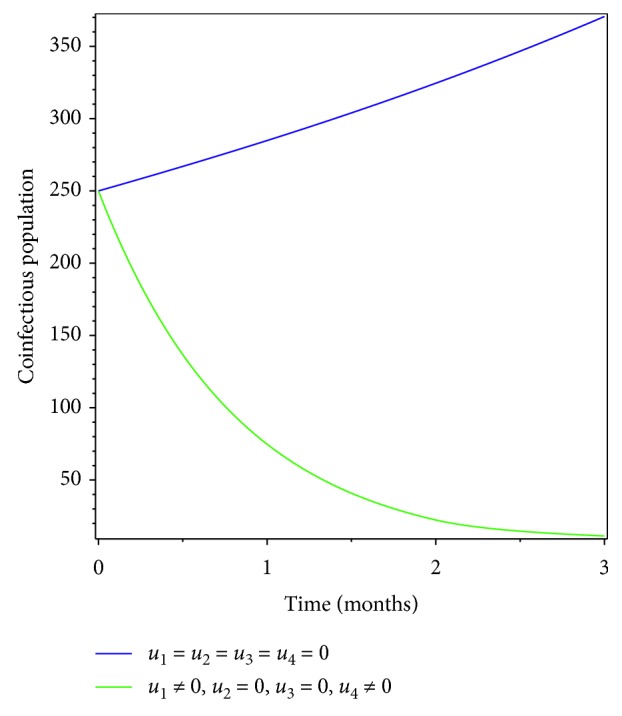
Effect of prevention of pneumonia and treatment of meningitis on coinfectious populations.

Here we investigated the effect of combination of prevention and treatment as the intervention strategy. Figures 4 and 5 show that prevention for pneumonia only and treatment for meningitis only contribute in controlling pneumonia-only infectious population, meningitis-only infectious population, and coinfectious population.

### 5.3. Control with Prevention Effort for Meningitis and Treatment Effort for Pneumonia

**Figure 6:**
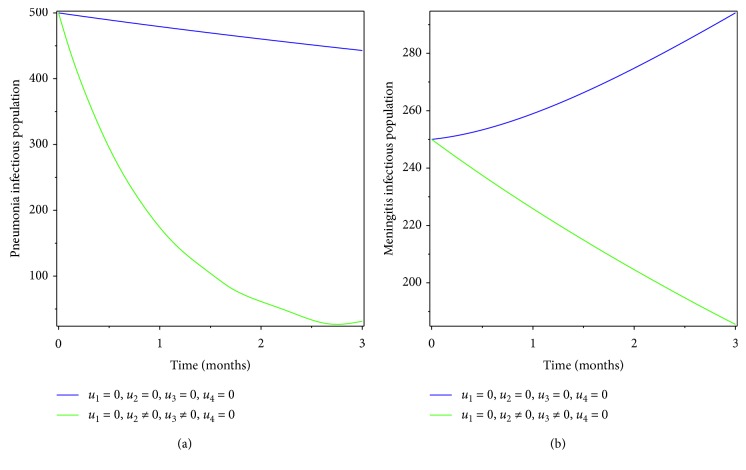
Simulations of optimality system with prevention of meningitis and treatment of pneumonia.

**Figure 7:**
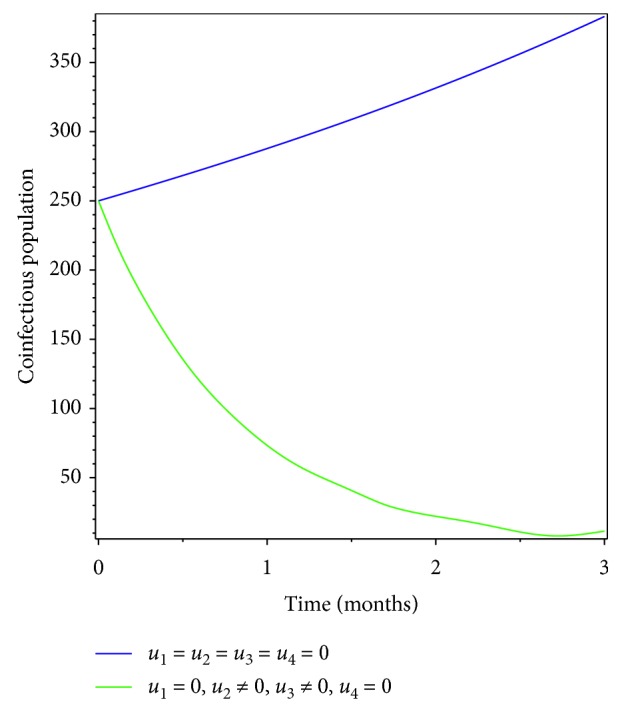
Effect of prevention of meningitis and treatment of pneumonia on coinfectious populations.

In this section, we used prevention of meningitis and treatment of pneumonia as the controlling mechanism. Figures 6 and 7 show that pneumonia and meningitis infectious populations and coinfectious populations are found to increase due to lack of intervention, but when meningitis prevention and pneumonia treatment are used as the intervention mechanism, the infectious population is found to decreaseat the specified time.

### 5.4. Control with Treatment Effort for Both Diseases

**Figure 8:**
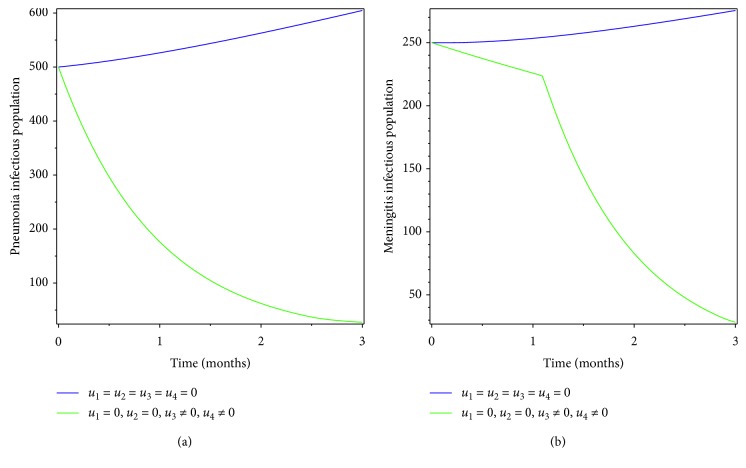
Simulations of optimality system with treatment effort.

**Figure 9:**
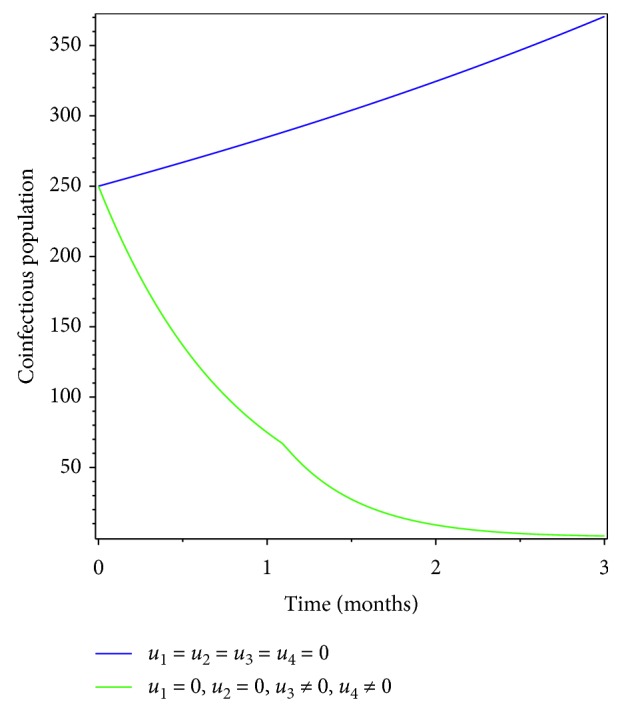
Effect of treating both diseases on coinfectious populations.

In this section, treatment of pneumonia and meningitis diseases is used as the controlling strategy. The results of the applications of the strategies are shown in Figures 8 and 9. From the figures, we see that the infectious population due to pneumonia and meningitis diseases as well as the coinfectious population is found to decrease due to treatment strategy for both diseases.

### 5.5. Control with All Intervention Strategies

**Figure 10:**
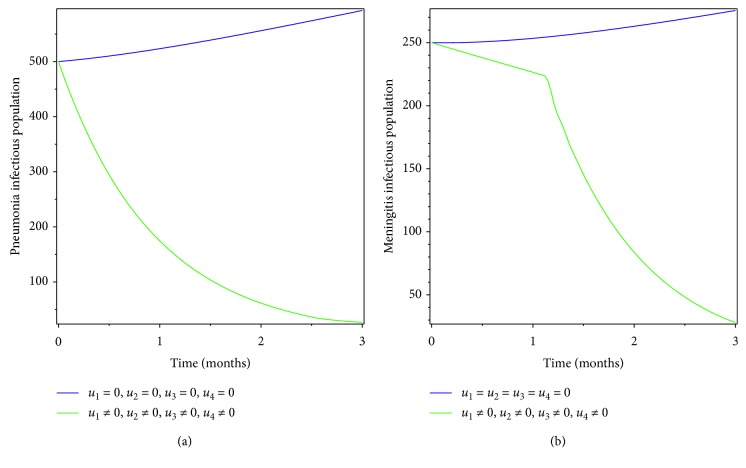
Simulations of optimality system with all interventions.

**Figure 11:**
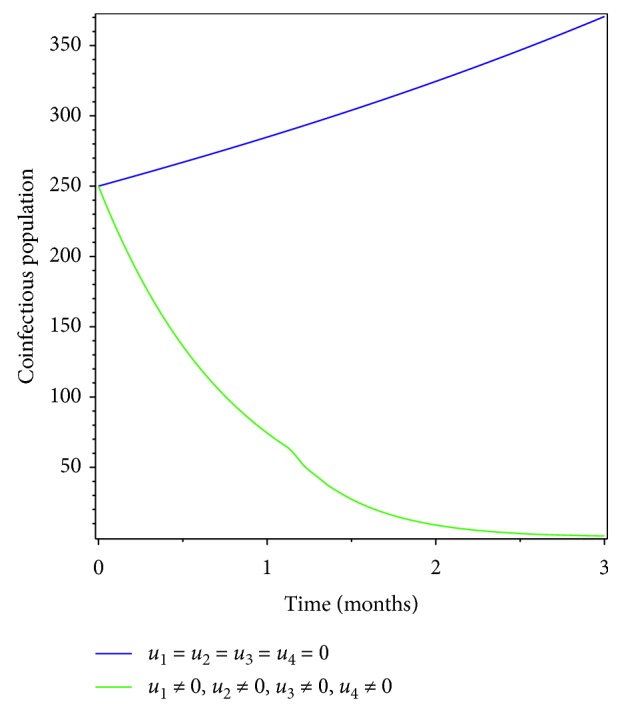
Effect of all control interventions on coinfectious populations.

In this section, we used all the four controlling strategies to tackle pneumonia, meningitis, and their coinfection. The results from Figures 10 and 11 show that the proposed intervention strategies are effective in bringing down the infectious population in the specified period of time.

## 6. Discussion and Conclusion

In Section 2, the basic model is described. The total system is subdivided into seven compartments by using ordinary differential equations. The qualitative behaviours including the invariant region, the positivity of solution, the disease-free equilibrium, basic reproduction number, analysis of disease-free equilibrium points, and checking the sensitivity of each parameter are presented in Section 3. In Section 4, the basic model is extended to optimal control by incorporating four controls such as prevention of pneumonia, prevention of meningitis, treatment of pneumonia, and treatment of meningitis. In this section, we characterized the optimal controls in terms of optimality system solutions. In Section 5, the optimality system is simulated by applying the Runge–Kutta forward-backward sweep method. For simulation of the optimality system, we proposed five strategies to check the effect of the controls. First, we considered prevention only for both diseases, and the result shows that applying prevention control has a great impact in bringing down the expansion of pneumonia, meningitis, and their coinfection in the specified period of time. The other strategies are prevention effort for pneumonia and treatment effort for meningitis, prevention effort for meningitis and treatment effort for pneumonia, treatment effort for both diseases, and using all interventions. We obtained that each of the listed strategies is effective in minimizing the expansion of pneumonia-only infectious population, meningitis-only infectious population, and coinfectious population in the specified period of time.

## Figures and Tables

**Table 1:** Parameter values of the model.

Parameter symbol	Value	Source
*δ*_1_	0.003–0.1	[7]
*δ*_2_	0.00904–0.99	[3]
*δ*_3_	0.01	Assumed
*a*	0.007–0.6	[9]
*b*	0.9	[3]
*α*_1_	0.006–0.5	Estimated
*α*_2_	0.002–0.2	Estimated
*σ*	0.1	[7]
*g*	0.5–1	[9]
*e*	0.5–1	[9]
*μ*	0.01	Assumed
*σ*_1_	0.9	Assumed
*σ*_2_	0.8	Assumed

## Data Availability

The data used to support the findings of this study are included within the article.
